# Sex-Related Differences in Impact on Safety of Pharmacogenetic Profile for Colon Cancer Patients Treated with FOLFOX-4 or XELOX Adjuvant Chemotherapy

**DOI:** 10.1038/s41598-019-47627-1

**Published:** 2019-08-08

**Authors:** Annamaria Ruzzo, Francesco Graziano, Francesca Galli, Fabio Galli, Eliana Rulli, Sara Lonardi, Monica Ronzoni, Bruno Massidda, Vittorina Zagonel, Nicoletta Pella, Claudia Mucciarini, Roberto Labianca, Maria Teresa Ionta, Irene Bagaloni, Enzo Veltri, Pietro Sozzi, Sandro Barni, Vincenzo Ricci, Luisa Foltran, Mario Nicolini, Edoardo Biondi, Annalisa Bramati, Daniele Turci, Silvia Lazzarelli, Claudio Verusio, Francesca Bergamo, Alberto Sobrero, Luciano Frontini, Mauro Magnani

**Affiliations:** 1 Department of Biomolecular Sciences, Università degli Studi di Urbino “Carlo Bo”, Urbino, Italy; 2 Azienda Ospedaliera “Ospedali Riuniti Marche Nord”, Pesaro, Italy; 3 Laboratory of Methodology for Clinical research, Department of Oncology, Istituto di Ricerche Farmacologiche Mario Negri IRCCS, Milano, Italy; 4 IOV- IRCCS, Padova, Italy; 5 Ospedale San Raffaele, Milano, Italy; 6 Azienda Ospedaliera Universitaria di Cagliari, P.O. Monserrato, Monserrato, Italy; 7 Azienda Ospedaliera S. Maria della Misericordia, Udine, Italy; 8 Ospedale “B. Ramazzini”, Carpi, Italy; 9 Ospedale Papa Giovanni XXIII, Bergamo, Italy; 10 Ospedale di Gaeta ASL Latina, Gaeta, Italy; 11 Ospedale degli Infermi di Biella, Biella, Italy; 12 Ospedale “Treviglio-Caravaggio”, Treviglio, Italy; 13 Azienda Ospedaliera Santa Maria degli Angeli, Pordenone, Italy; 14 Azienda Ospedaliera Ospedale “Cervesi”, Cattolica, Italy; 15 Ospedale “F. Renzetti”, Lanciano, Italy; 16 Azienda Ospedaliera Fatebenefratelli, Milano, Italy; 17 AUSL Ospedale di Ravenna, Ravenna, Italy; 18 Azienda Ospedaliera di Cremona, Cremona, Italy; 19 Ospedale di Saronno, Saronno, Italy; 20 Azienda Ospedaliera “Ospedale San Martino”, Genova, Italy; 21 Fondazione GISCAD, Vanzago, Italy

**Keywords:** Predictive markers, Colon cancer

## Abstract

Polymorphisms contribute to inter-individual differences and show a promising predictive role for chemotherapy-related toxicity in colon cancer (CC). TOSCA is a multicentre, randomized, non-inferiority, phase III study conducted in high-risk stage II/stage III CC patients treated with 6 vs 3 months of FOLFOX-4 or XELOX adjuvant chemotherapy. During this post-hoc analysis, 218 women and 294 men were genotyped for 17 polymorphisms: TYMS (rs34743033, rs2853542, rs11280056), MTHFR (rs1801133, rs1801131), ERCC1 (rs11615), XRCC1 (rs25487), XRCC3 (rs861539), XPD (rs1799793, rs13181), GSTP1 (rs1695), GSTT1/GSTM1 (deletion +/−), ABCC1 (rs2074087), and ABCC2 (rs3740066, rs1885301, rs4148386). The aim was to assess the interaction between these polymorphisms and sex, on safety in terms of time to grade ≥3 haematological (TTH), grade ≥3 gastrointestinal (TTG) and grade ≥2 neurological (TTN) toxicity. Interactions were detected on TTH for rs1801133 and rs1799793, on TTG for rs13181 and on TTN for rs11615. Rs1799793 GA genotype (p = 0.006) and A allele (p = 0.009) shortened TTH in men. In women, the rs11615 CC genotype worsened TTN (co-dominant model p = 0.008, recessive model p = 0.003) and rs13181 G allele improved the TTG (p = 0.039). Differences between the two sexes in genotype distribution of rs1885301 (p = 0.020) and rs4148386 (p = 0.005) were found. We highlight that polymorphisms could be sex-specific biomarkers. These results, however, need to be confirmed in additional series.

## Introduction

Standard regimens of adjuvant chemotherapy for patients with colon cancer (CC) include oxaliplatin combined with bolus/infusional 5-fluorouracil (FOLFOX) or capecitabine (XELOX)^1^. The efficacy of platinum-based drugs is often compromised because of the substantial risk for severe toxicities, including neurotoxicity. Many patients experience side effects at some point during treatment and the most frequently reported adverse events of these regimens in randomized adjuvant trials in Western populations are neutropenia (≥ grade 3 in 40% to 56% of patients), neurotoxicity (≥ grade 3 in 10% to 20% of patients), and diarrhea (≥ grade 3 in 10% to 15% of patients)^2,3^. Adverse drug events (ADEs) are responsible for treatment delay, reduction, cessation, or, in a minority of cases, the death of a patient.

Increasing evidence has shown that sex differences exist in ADEs^4^. Distinguishing gender (psychosocial-cultural, how people perceive themselves and others) and sex (biological, including sex chromosomes, gene expression, hormone levels, and reproductive/sexual anatomy) differences, both sex and gender have an effect on how an individual selects, responds to, metabolizes, and adheres to a particular therapy^5,6^.

We published a pharmacogenetic ancillary study^2^ of the TOSCA trial^7,8^, aiming to assess the impact on toxicity of selected polymorphisms described on 11 genes involved in DNA repair and drug metabolism. The study whose results are here reported was inspired by the growing interest in gender medicine focused on the impact of sex on the management of the diseases. The analysis was aimed to investigate potential differences in the impact of the genetic variations on toxicity and efficacy outcomes in a subgroup of women and men from the TOSCA ancillary study.

## Material and Methods

As reported in Lonardi *et al*.^7^, TOSCA is a phase III, randomized, open-label, non-inferiority, multicenter trial conducted in 130 Italian centers and involving 3759 patients with resected CC located >12 cm from the anal verge by endoscopy and/or above the peritoneal reflection at surgery. No gross or microscopic evidence of residual disease after surgery was allowed. The study was conducted in accordance with the Declaration of Helsinki and adhered to Good Clinical Practice guidelines. Approval was obtained from local Ethics Committee for each participating site (see supplementary information file), and all patients provided written informed consent to the study. Other details on TOSCA trial was described elsewhere^7,8^. Patients eligible for the TOSCA trial were asked to provide additional written informed consent to be enrolled in pharmacogenetic studies. The TOSCA ancillary study enrolled 218 women and 294 men, from 26 Italian centers, between 2007 and 2011. Since data about sex/gender were not collected consistently, it is not possible to distinguish between sex and gender in our analyses. Therefore, the terms sex and gender could be used interchangeably.

### Assessment and management of chemotherapy toxicity

Selected hematologic and non-hematologic toxicities (anemia, leukopenia, neutropenia, thrombocytopenia, asthenia, diarrhea, mucositis, stomatitis, vomiting, nausea, hepatic toxicity, skin toxicity, neurotoxicity) were assessed at the start of each cycle using Common Toxicity Criteria for Adverse Events (CTCAE), version 2.0. All adverse events during the course of the study were monitored and reported. As previously reported in Ruzzo *et al*.^2^, toxicity was managed as follows: in case of grade ≥3 or persistent grade 2 hematologic toxicity, the dose of all drugs was reduced by 25%. In case of grade ≥3 non-hematologic toxicity, the dose of the related drugs was reduced by 50%. In case of grade ≥3 or persistent grade 2 neurotoxicity, the oxaliplatin dose was reduced by 20%. Oxaliplatin was permanently discontinued if grade ≥2 neurosensory symptoms persisted between cycles. Once a dose has been reduced because of toxicity, there was no dose re-escalation in subsequent cycle.

### Molecular and genetic assessments

The protocol used to assess the patients’ genotypes has been extensively reported elsewhere^6^. The genetic variations analyzed are the following: TYMS (rs 34743033, rs2853542, rs11280056), MTHFR (rs1801133, rs1801131), ERCC1 (rs11615), XRCC1 (rs25487), XRCC3 (rs861539), XPD (rs1799793 and rs13181), GSTP1 (rs1695), GSTT1/GSTM1 (delection +/−), ABCC1 (rs2074087) and ABCC2 (rs3740066, rs1885301, rs4148386). These genes and polymorphisms were selected as being potentially predictive of 5-fluorouracil (5-FU) or oxaliplatin toxicity in CC patients^2,9–14^.

### Statistical analysis

Potential differences between women and men in the effects of the selected polymorphism on toxicity in terms of time to grade ≥3 hematological toxicity (except anemia, TTH), time to grade ≥3 anemia (TTA), time to grade ≥3 gastrointestinal toxicity (TTG) and time to grade ≥2 neurological toxicity (TTN) were explored. Hematological toxicity includes leukopenia, febrile and non-febrile neutropenia and thrombocytopenia; gastrointestinal toxicity includes diarrhea, nausea, vomiting, stomatitis and mucositis. Finally, neurological toxicity includes ototoxicity, central neurotoxicity and paresthesia/dysesthesia. TTH, TTA, TTG and TTN were defined as the time from the date of randomization to the date of first specific toxicity. Subjects without such a toxicity event at the time of analysis were censored at the date they were last known to be event-free while on treatment. Time-to-toxicity was selected instead of the incidence of toxicity as the endpoint in order to improve statistical power and capture potential clinically meaningful differences in time to the onset of toxicity, especially in the case of few observations (due to the rarity of some genotype), as suggested by Thanarajasingam *et al*.^15^.

To reduce the number of comparison, each polymorphism (Table 1) was analyzed according to the probable biological function of the relative gene and the clinical annotations reported in the PharmGKB database (www.pharmgkb.org). Therefore, the effect on TTH, TTA and TTG was investigated only for genetic variations on TYMS, MTHFR, XPD, XRCC3, GSTP1, GSTT1/GSTM1, ABCC1 and ABCC2 whereas the effect on TTN was investigated only for genetic variations on MTHFR, ERCC1, XRCC1, XPD, GSTP1, GSTT1/GSTM1, ABCC1 and ABCC2 genes.

**Table 1:** Genes, genetic variations, genotype and allele frequencies in women and men.

Gene (site) ID number	Type of variation	Genotype (amino acid change)	N°pts W/M	W/M genotype N° patients (genotype frequency W/M)				Allele Frequency W/M	
				AA	Aa	aa	*p*-value*	A	a
TYMS (5′UTR) rs34743033	VNTR**§**	3 R > 2 R	217/294	67 (0.31)/106 (0.36)	108 (0.50)/130 (0.44)	42 (0.19)/58 (0.20)	0.4017	0.56/0.58	0.44/0.42
TYMS (5′UTR) rs2853542	SNP**§**	G > C in 3 R	217/294	117 (0.54)/146 (0.50)	—	100 (0.46)/148 (0.50)	0.3412	0.54/0.50	0.46/0.50
TYMS (3′UTR) rs11280056	6 bp deletion	Insertion/Deletion	217/294	82 (0.38)/105 (0.36)	101 (0.47)/135 (0.46)	34 (0.16)/54 (0.18)	0.7098	0.60/0.59	0.40/0.41
MTHFR (exon 4) rs1801133	SNP	C > T (Ala222Val)	217/293	71 (0.33)/90 (0.31)	101 (0.47)/148 (0.51)	45 (0.21)/55 (0.19)	0.6683	0.56/0.56	0.44/0.44
MTHFR (exon 7) rs1801131	SNP	A > C (Glu429Ala)	217/293	109 (0.50)/142 (0.48)	88 (0.41)/125 (0.43)	20 (0.09)/26 (0.09)	0.8922	0.70/0.70	0.30/0.30
ERCC1 (exon 4) rs11615	SNP	T > C (Asn118Asn)	218/294	86 (0.39)/111 (0.38)	100 (0.46)/128 (0.44)	32 (0.15)/55 (0.19)	0.4862	0.62/0.60	0.38/0.40
XRCC1 (exon 10) rs25487	SNP	G > A (Gln399Arg)	215/291	90 (0.42)/119 (0.41)	97 (0.45)/142 (0.49)	28 (0.13)/30 (0.10)	0.5551	0.64/0.65	0.36/0.35
XPD (exon 10) rs1799793	SNP	G > A (Asp312Asn)	210/285	85 (0.40)/125 (0.44)	89 (0.42)/127 (0.45)	36 (0.17)/33 (0.12)	0.2070	0.62/0.66	0.38/0.34
XPD (exon 23) rs13181	SNP	T > G (Lys751Gln)	214/294	78 (0.36)/113 (0.38)	99 (0.46)/137 (0.47)	37 (0.17)/44 (0.15)	0.7586	0.60/0.62	0.40/0.38
XRCC3 (exon 7) rs861539	SNP	C > T (Thr241Met)	213/291	63 (0.30)/108 (0.37)	105 (0.49)/138 (0.47)	45 (0.21)/45 (0.15)	0.1132	0.54/0.61	0.46/0.39
GSTPI (exon 5) rs1695	SNP	A > G (Ile105Val)	217/293	94 (0.43)/150 (0.51)	104 (0.48)/121 (0.41)	19 (0.09)/22 (0.08)	0.2123	0.67/0.72	0.33/0.28
GST-T1**‡**	Deletion	Yes/No	217/294	176 (0.81)/243 (0.83)	—	41 (0.19)/51 (0.17)	0.6528	0.81/0.83	0.19/0.17
GST-M1**‡**	Deletion	Yes/No	217/294	112 (0.52)/150 (0.51)	—	105 (0.48)/144 (0.49)	0.8946	0.52/0.51	0.48/0.49
ABCC1 (intron) rs2074087	SNP	G > C	202/277	144 (0.71)/197 (0.71)	54 (0.27)/73 (0.26)	4 (0.02)/7 (0.03)	0.9236	0.85/0.84	0.15/0.16
ABCC2 (exon 28) rs3740066	SNP	G > A (Ile1324Ile)	216/293	86 (0.40)/102 (0.35)	99 (0.46)/145 (0.49)	31 (0.14)/46 (0.16)	0.5122	0.63/0.60	0.37/0.40
ABCC2 (5′flank) rs1885301	SNP	G > A	217/285	81 (0.37)/76 (0.27)	89 (0.41)/149 (0.52)	47 (0.22)/60 (0.21)	0.0203	0.58/0.53	0.42/0.47
ABCC2 (intron) rs4148386	SNP	A > G	217/294	85 (0.39)/79 (0.27)	87 (0.40)/157 (0.53)	45 (0.21)/58 (0.20)	0.0050	0.59/0.54	0.41/0.46

A: major allele frequency; a: minor allele frequency; VNTR: variable number of tandem repeats; SNP: single nucleotide polymorphism; bp: base pair; pts: patients; W/M: women/men;

**§**TYMS VNTR: is a tandem repeat polymorphism, results are stated as three copies of the repeat (AA) or two copies of the repeat (aa). The VNTR polymorphism is reanalyzed according to a SNP in 3 R carriers.

**‡**GST -T1 and -M1 are deletion polymorphisms, resulte are stated as the number of patients with at least one copy of the gene (AA) vs patients with homozygous gene deletion (aa). *Chi-squared test women vs men.

Moreover, interaction tests were performed to detect different effects of polymorphisms on each endpoint in women and men and subgroups analyses according to sex were done only for polymorphisms for which such a difference were significantly demonstrated. Lastly, only these selected polymorphisms were analyzed to test potential differences between women and men on efficacy in terms of relapse free survival (RFS) and overall survival (OS). RFS was defined as the time from the date of randomization to the earlier of the date of relapse or death from any cause. Patients alive without relapse while on study were censored at the last disease assessment date. OS was defined as the time from the date of randomization to date of death from any cause. Patients who remained alive while on study were censored at the date they were last known to be alive. Separate Cox proportional hazard models were used to investigate the interaction between each polymorphism and sex for each toxicity. Separate sex-specific Cox models were used to assess the effects of each selected polymorphism on clinical endpoints. Results, adjusted for treatment duration (3 or 6 months), were provided as the hazard ratio (HR) with 95% confidence interval (95% CI). Dose reduction was included in each model as a dichotomous time-dependent covariate. This variable can vary over time, assuming value 1 in case of dose reduction for any cause. Since the purpose of this analysis is hypothesis-generating, no correction for multiple testing was applied. Anyway, to test the robustness of the results obtained by the above-specified analyses, logistic models, adjusted for treatment duration and dose reduction occurred before the specific toxicity, were also performed. Patients were categorized in three genotype groups: carriers of the homozygous wild type or more frequent genotype (AA), heterozygous (Aa), and homozygous variant or less frequent genotype (aa). The effect of variant on endpoints was analyzed according to three genetic models: (1) in the co-dominant model, each effect of Aa and aa genotypes compared to AA were estimated; (2) assuming an equal effect of the presence of one or two mutant alleles, the dominant model pooled patients with Aa or aa variants and compared them to the patients with AA genotype; (3) hypothesizing that the presence of only one mutant allele does not significantly impact clinical endpoints, the recessive model tested the effect of the aa genotype to the pooled Aa or AA genotypes. Hardy-Weinberg equilibrium (HWE) was tested separately in both sexes.

Differences between women and men in term of baseline characteristics were investigated using the chi-squared test (or Fisher’s exact test where needed) for categorical variables, and t-test for continuous variables. All reported p-values were two-sided with *p* < 0.05 value considered statistically significant. Analyses were performed with SAS 9.4 (SAS Institute, Cary, NC) and the SNPStats package^16^.

## Results

The allele and genotype frequencies in 218 women and 294 men are reported in Table 1. A different distribution between women and men for the ABCC2 rs1885301 and rs4148386 genotypes was observed (*p* = 0.0203 and *p* = 0.0050, respectively) (Table 1), confirmed by the HWE departure in women for these two polymorphisms (*p* = 0.0191 and *p* = 0.0122, respectively). Demographic, clinical and tumor characteristics are listed by sex in Table 2 and in Table 3; a significant higher proportion of women with right-sited CC compared to men is shown in Table 2, 40.0% vs 34.7% respectively (*p* = 0.0426,). Comparison in terms of baseline characteristics between our sample and the TOSCA population were provided as supplementary materials (Tables S1 and S2).

**Table 2:** Demographic and clinical characteristics of patients.

	Men N = 294	Women N = 218	Overall N = 512	T-test or Chi-squared test *p*-value
Age				0.1074
Mean (SD)	63.8 (9.3)	62.5 (9.8)	63.3 (9.5)	
Median (Q1–Q3)	64.5 (58.7–70.9)	63.2 (56.4–69.8)	64.0 (57.4–70.7)	
Min–Max	25.1–82.3	34.3–81.9	25.1–82.3	
Performance status - n (%)				0.7473
0	283 (96.3)	211 (96.8)	494 (96.5)	
1	11 (3.7)	7 (3.2)	18 (3.5)	
Tumor site				0.4391
Single site	279 (94.9)	210 (96.3)	489 (95.5)	
Multiple site	15 (5.1)	8 (3.7)	23 (4.5)	
Single site specification - n (%)				0.4849
Ascending colon	74 (26.5)	64 (30.5)	138 (28.2)	
Hepatic flexure	13 (4.7)	14 (6.7)	27 (5.5)	
Trasverse colon	15 (5.4)	17 (8.1)	32 (6.5)	
Splenic flexure	13 (4.7)	11 (5.2)	24 (4.9)	
Descending colon	46 (16.5)	27 (12.9)	73 (14.9)	
Sigmoid colon	77 (27.6)	54 (25.7)	131 (26.8)	
Sigmoid-rectum colon	41 (14.7)	23 (11.0)	64 (13.1)	
Missing	15	8	23	
Tumor side - n (%)				0.0426
Right sides	102 (34.7)	96 (44.0)	198 (38.7)	
Left sides	178 (60.5)	115 (52.8)	293 (57.2)	
Multiple side*	14 (4.8)	7 (3.2)	21 (4.1)	

*This category includes patients with both right and left tumor sides. The statistical test was performed excluding patients with multiple sided tumor.

**Table 3:** Tumor characteristics.

	Men N = 294	Women N = 218	Overall N = 512	Chi-squared test *p*-value
Histology - n (%)				0.2677^A^
Adenocarcinoma	249 (84.7)	192 (88.1)	441 (86.1)	
Mucoid adenocarcinoma	42 (14.3)	23 (10.6)	65 (12.7)	
Ring cell carcinoma	1 (0.3)	2 (0.9)	3 (0.6)	
Medullary carcinoma	2 (0.7)	0 (0.0)	2 (0.4)	
Other	0 (0.0)	1 (0.5)	1 (0.2)	
Histology categorization - n (%)				0.4324^A^
Adenocarcinoma	249 (84.7)	192 (88.1)	441 (86.1)	
Mucoid adenocarcinoma	42 (14.3)	23 (10.6)	65 (12.7)	
Other	3 (1.0)	3 (1.4)	6 (1.2)	
T stage - n (%)				0.9437
Tx	1 (0.3)	0 (0.0)	1 (0.2)	
T1	6 (2.0)	6 (2.8)	12 (2.3)	
T2a	8 (2.7)	7 (3.2)	15 (2.9)	
T2b	9 (3.1)	7 (3.2)	16 (3.1)	
T3	221 (75.2)	164 (75.2)	385 (75.2)	
T4	49 (16.7)	34 (15.6)	83 (16.2)	
N stage - n (%)				0.3398
N0	100 (34.0)	85 (39.0)	185 (36.1)	
N1	137 (46.6)	100 (45.9)	237 (46.3)	
N2	57 (19.4)	33 (15.1)	90 (17.6)	
Clinical stage - n (%)				0.2464
II	100 (34.0)	85 (39.0)	185 (36.1)	
III	194 (66.0)	133 (61.0)	327 (63.9)	
Clinical stage subgrups - n (%)				0.2734
II	100 (34.0)	85 (39.0)	185 (36.1)	
III low risk	121 (41.2)	91 (41.7)	212 (41.4)	
III high risk	73 (24.8)	42 (19.3)	115 (22.5)	
Grade - n (%)				0.3639^A^
GX	1 (0.3)	3 (1.4)	4 (0.8)	
G1	25 (8.6)	13 (6.0)	38 (7.5)	
G2	172 (59.1)	135 (62.5)	307 (60.6)	
G3	93 (32.0)	65 (30.1)	158 (31.2)	
Missing	3	2	5	
Chemotherapy taken during the TOSCA trial - n (%)				0.6404
Folfox-4 (6 months)	100 (34.0)	86 (39.4)	186 (36.3)	
Xelox (24 weeks)	43 (14.6)	28 (12.8)	71 (13.9)	
Folfox-4 (3 months)	110 (37.4)	77 (35.3)	187 (36.5)	
Xelox (12 weeks)	41 (13.9)	27 (12.4)	68 (13.3)	

^A^Fisher test *p*-value.

**Figure 1:**
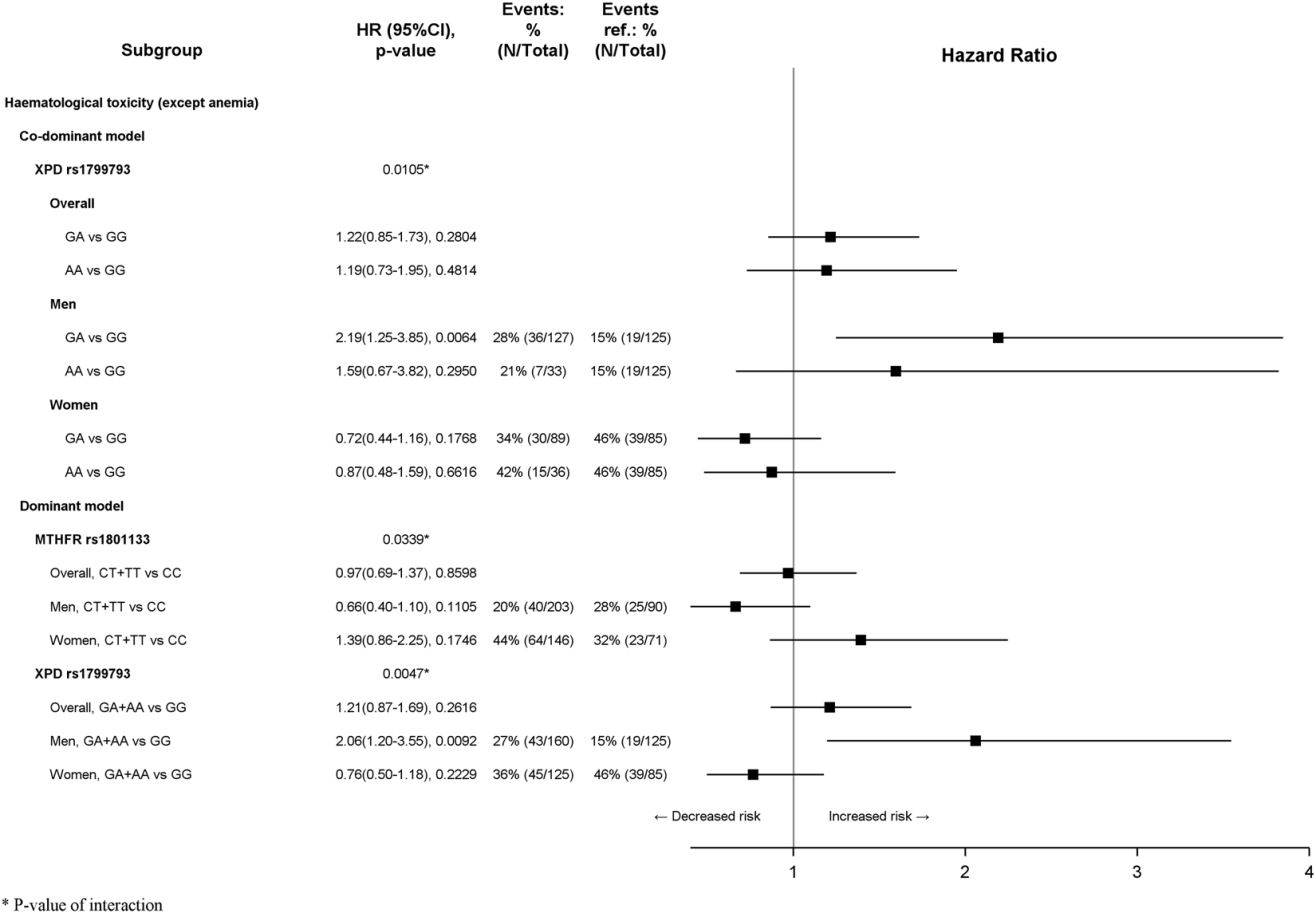
Subgroup analysis according to sex for time to haematological toxicity (TTH).

**Figure 2:**
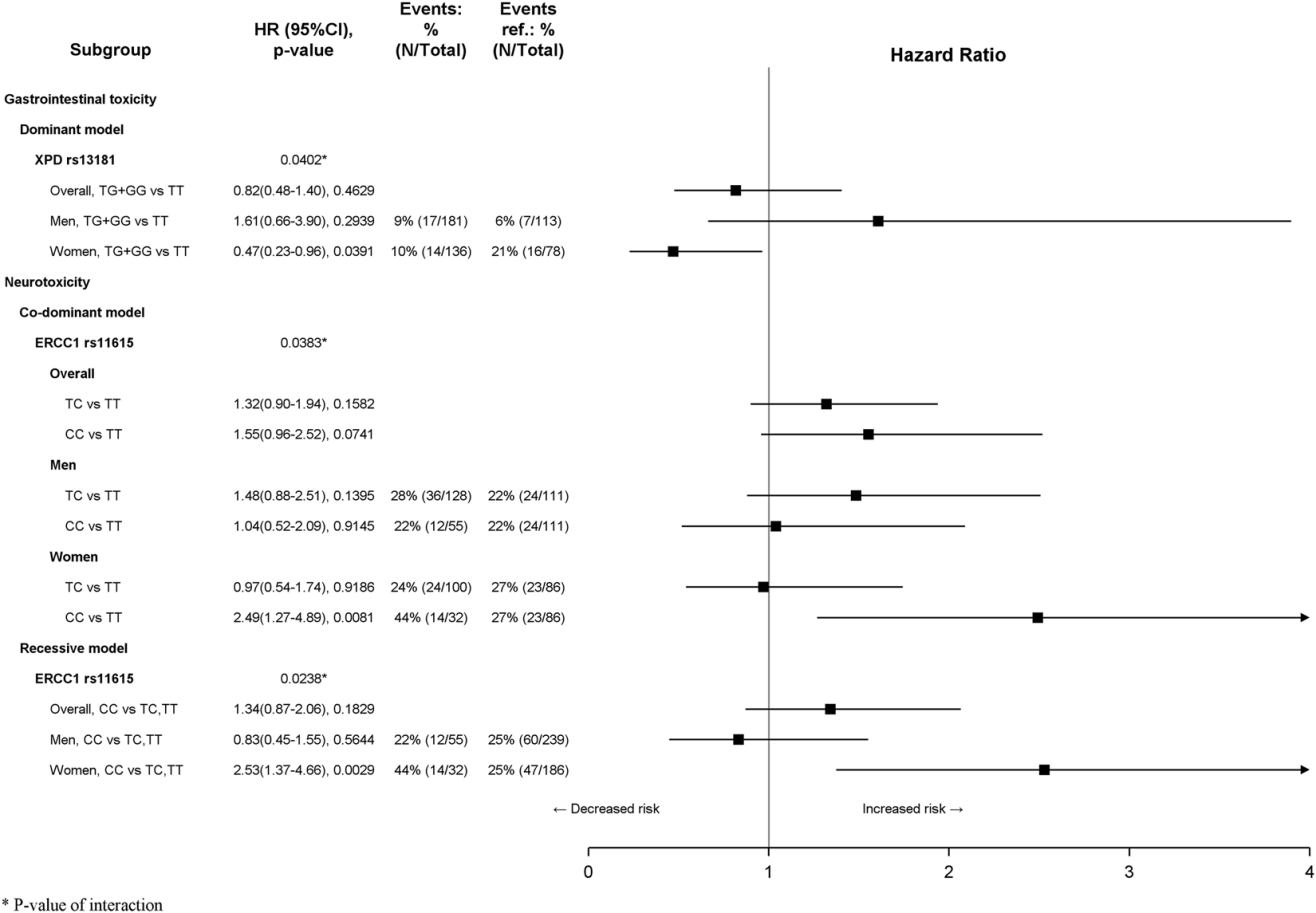
Subgroup analysis according to sex for time to gastrointestinal (TTG) and neurotoxicity (TTN).

After a median follow-up of 74.5 months (75.5 in women and 73.7 in men, *p* = 0.9260), 152 (29.7%) patients experienced grade ≥3 hematological events, 2 (0.4%) experienced grade ≥3 anemia, 55 (10.7%) experienced grade ≥3 gastrointestinal toxicity and 133 (26.0%) experienced grade ≥2 neurotoxicity. Moreover, 71 (13.9%) deaths and 106 (20.7%) relapses or deaths were recorded. A significant sex difference in the proportion of patients who experienced grade ≥3 hematological toxicity (39.9% of women and 22.1% of men, *p* < 0.0001) and grade ≥3 gastrointestinal toxicity (14.2% of women and 8.2% of men, *p* = 0.0286) was found (Table 4). Due to the low number of anemia events, no analyses were performed on TTA. Interaction between sex and polymorphisms was detected on TTH for XPD rs1799793 (co-dominant and dominant model, *p*_*interaction*_ = 0.0105 and *p*_*interaction*_ = 0.0047, respectively) and MTHFR rs1801133 (dominant model, *p*_*interaction*_ = 0.0339). Moreover, significant interaction with sex was found on TTG for XPD rs13181 (dominant model, *p*_*interaction*_ = 0.0402) and on TTN for ERCC1 rs11615 (co-dominant and recessive model, *p*_*interaction*_ = 0.0383 and *p*_*interaction*_ = 0.0238, respectively). Results of subgroup analysis by sex on TTH are summarized in Fig. 1. No significant effects of genetic variants in women were detected. In men, according to co-dominant model, the XPD rs1799793 GA genotype was associated with a worse TTH (HR 2.19; 95% CI 1.25 to 3.85; *p* = 0.0064); more generally, according to dominant model, the presence of at least one XPD rs1799793 A allele worsened the TTH (HR 2.06; 95% CI 1.20 to 3.55; *p* = 0.0092). MTHFR rs1801133 did not reach statistical significance in women nor in men. Results of subgroup analysis by sex on TTG and TTN are summarized in Fig. 2. No significant effects of genetic variants in men were detected. In women, XPD rs13181 was associated with TTG, whereas ERCC1 rs11615 was associated with TTN. In detail, according to the dominant model, the presence of at least one XPD rs13181 G allele was associated with improved TTG (HR 0.47; 95% CI 0.23 to 0.96; *p* = 0.0391). In women, the ERCC1 rs11615 CC genotype was associated with a worse TTN according both to co-dominant model (HR 2.49; 95% CI 1.27 to 4.89; *p* = 0.0081) and to recessive model (HR 2.53; 95% CI 1.37 to 4.66; *p* = 0.0029). Finally, no interactions between sex and these polymorphisms on efficacy endpoints were detected, therefore no subgroup analyses were performed. All significant results were confirmed by means of logistic regression, except the interaction between the ERCC1 rs11615 and sex according to the co-dominant model (*p*_*interaction*_ = 0.0518, Supplementary Fig. S1).

**Table 4:** Toxicity and Clinical Events.

	Men N = 294	Women N = 218	Overall N = 512	Chi squared test *p*-value
**Grade ≥ 3 heamatological toxicity (except anemia) - n (%)**	65 (22.1)	87 (39.9)	152 (29.7)	<0.0001
Grade ≥ 3 leukopenia - n (%)	6 (2.0)	5 (2.3)	11 (2.1)	
Grade ≥ 3 febrile neutropenia - n (%)	4 (1.4)	6 (2.8)	10 (2.0)	
Grade ≥ 3 non-febrile neutropenia - n (%)	62 (21.1)	81 (37.2)	143 (27.9)	
Grade ≥ 3 thrombocytopenia - n (%)	1 (0.3)	4 (1.8)	5 (1.0)	
**Grade ≥ 3 anemia - n (%)**	1 (0.3)	1 (0.5)	2 (0.4)	—
**Grade ≥ 3 gastrointestinal toxicity - n (%)**	24 (8.2)	31 (14.2)	55 (10.7)	0.0286
Grade ≥ 3 diarrhea - n (%)	14 (4.8)	20 (9.2)	34 (6.6)	
Grade ≥ 3 nausea - n (%)	6 (2.0)	8 (3.7)	14 (2.7)	
Grade ≥ 3 vomiting - n (%)	5 (1.7)	6 (2.8)	11 (2.1)	
Grade ≥ 3 stomatitis - n (%)	1 (0.3)	1 (0.5)	2 (0.4)	
Grade ≥ 3 mucositis - n (%)	1 (0.3)	3 (1.4)	4 (0.8)	
**Grade ≥ 2 neurotoxicity - n (%)**	72 (24.5)	61 (28.0)	133 (26.0)	0.3730
Grade ≥ 2 ototoxicity - n (%)	1 (0.3)	0 (0.0)	1 (0.2)	
Grade ≥ 2 central neurotoxicity - n (%)	9 (3.1)	7 (3.2)	16 (3.1)	
Grade ≥ 2 paresthesia/dysesthesia - n (%)	63 (21.4)	55 (25.2)	118 (23.0)	
Relapse - n (%)	50 (17.0)	32 (14.7)	82 (16.0)	0.4776
Death - n (%)	45 (15.3)	26 (11.9)	71 (13.9)	0.2739
Relapse or death - n (%)	67 (22.8)	39 (17.9)	106 (20.7)	0.1761

## Discussion

The majority of cytotoxic drugs have a dose-related effect and a narrow therapeutic index; thus, dose selection is crucial as even small dose variations can lead to significant toxicity in some patients and to under-dosing in others. Nevertheless chemotherapies are still mostly chosen based on age, height and body mass calculated as BSA (Body Surface Area), sometimes with the addition of TDM (Therapeutic Drug Monitoring)^17,18^. However, these characteristics do not entirely equalize inter-individual variations dependent on physiological, genetic and environmental factors (e.g. drug-drug interactions and drug-food interactions)^19^. The objective of our analysis was to investigate potential differences between men and women in the impact of selected genetic variations on fluoropyrimidine/oxaliplatin toxicity. Results show that genetic variants can predict toxicity to fluoropyrimidine/oxaliplatin differently in women and men affected by CC, supporting the hypothesis that sex has a role on molecular etiology and clinical outcomes. Specifically, XPD rs1799793 and MTHFR rs1801133 seem to have a different impact in men and women on time to heamatological toxicity, XPD rs13181 on time to gastrointestinal toxicity and ERCC1 rs11615 on neurotoxicity. ERCC1 and XPD genes are part of the nucleotide excision repair (NER) pathway, which repairs lesions induced by platinum-based chemotherapies. ERCC1 rs11615 T > C is associated with diminished expression levels of mRNA and protein with functional consequences in the repair of cisplatin DNA lesions, while XPD rs1799793 G > A alters the protein activity^20^. Even if ERCC1 rs11615 T > C and XPD rs1799793 G > A are on autosomal chromosomes, therefore shared by both sexes, their function could lie under a gene regulation different in the two sexes^21,22^. In other words, differences in gene regulation between women and men, rather than gene content, underlie most phenotypic sexual dimorphism, including sex-specific effects on human diseases, such as cancer, and probably other measurable phenotypes, including responses to therapies^23,24^. Also genetic mechanisms other than gene regulation (e.g., imprinting), might contribute to sexual dimorphism in quantitative phenotypes^24–26^.

In 2017, we published a pharmacogenetic study^27^ aimed to investigate the impact of DPYD genetic variants on fluoropyrimidine-related toxicity in the same group of TOSCA patients here analyzed. The DPYD genetic variants associated to toxicity are very rare. No differences between the two sexes were found, but we cannot exclude that these results were due to the low number of events.

In our sample, a different ABCC2 rs1885301 and rs4148386 genotype distribution between the two sexes was observed (*p* = 0.0203 and *p* = 0.0050, respectively). More in details we found a higher percentage of women carrying the homozygous rs1885301 GG and rs4148386 AA compared to men (37% vs 22% and 39% vs 21%, respectively), confirmed by the departure from HWE in women for these two polymorphisms (*p* = 0.018 and *p* = 0.011, respectively). ABCC2 (ATP Binding Cassette Subfamily C member 2), also known as MRP2 (Multidrug resistance-associated protein 2), is highly expressed in gut and localized to the apical plasma membrane of the enterocytes^28–30^. ABCC2, together with other ABC transporters, carries different substrates, both helpful and toxic, such as flavonoids and phytoestrogens, short chain fatty acids obtained through bacterial degradation of dietary fibres, carcinogens released by baked food, dietary fatty acids inducing pro- and anti-inflammatory signaling molecules. Therefore, ABCC2 contributes to extrude harmful substrates from the intestinal cells, reducing the absorption from the diet, limiting intestinal and systemic exposure^28–30^. Different studies reported that the increased ABCC2 gene expression is an early event during the transition from colorectal adenoma to carcinoma, and that the ABCC2 expression level seems to be regulated by sex hormones^28–31^. Moreover, Nguyen *et al*.^32^ demonstrated that the presence of rs1885301 G allele increased ABCC2 promoter activity compared to A allele. So, the higher frequency of rs1885301 GG genotype, found in women patients, could be explained by a synergistic effect between the decreased extrogen protection (due to menopause) against the CC, and the higher expression of ABCC2 due to GG genotype. In fact, as reported in Table 2, the mean age of women patients was 62.5.

In addition, the ABCC2 rs4148386 genotypes frequency were distributed differently in women compared to men (*p* = 0.0050), probably because the rs4148386 A allele is in linkage disequilibrium with rs1885301 G allele, as reported in “1000 genomes” (http://phase3browser.1000genomes.org/index.html) database for Italian (TSI) population, although a role of this polymorphism in colorectal carcinogenesis cannot be excluded.

In accord with several studies^33–37^ we found a higher proportion of women with right-sided CC than men, as shown in Table 2 (*p* = 0.0426). Since we found that both ABCC2 rs1885301 and rs4148386 polymorphisms had the HWE departure in women, we investigated potential differences in the ABCC2 genotypes distribution between sexes by tumor side. We found significant differences in the distribution of the ABCC2 genotypes between men and women in the subgroup of patients with right side tumor (rs1885301 G > A *p* = 0.0076 and rs4148386 A > G *p* = 0.0056 for ABCC2, respectively), while such a differences were not detected in the subgroup of patients with left side tumor (rs1885301 G > A *p* = 0.2393 and rs4148386 A > G *p* = 0.0668, respectively).

As showed in Table 4, haematological toxicity was more frequent in women (p < 0.0001), in particular grade ≥3 neutropenia. This is consistent with literature that reports women have higher risks of chemotherapy-induced neutropenia compared with men patients^38^ and so some authors propose that cutoff values for neutropenia should be re-established according to sex^39^.

Gender Medicine studies how diseases differ between women and men in terms of prevention, clinical signs, therapeutic approach, prognosis, predictability, psychological and social impact. It is conspicuous that in the era of personalized medicine the patients sex/gender is still quite undervalued.

Despite the evidence that there are physical and physiological differences between women and men, drug safety is rarely considered differently by sex in clinical treatment and the Food and Drug Administration (FDA) does not still require phase II clinical studies to compare dose and efficacy in the two sexes.

To date, this is one of few pharmacogenetic studies that mainly aims to assess how sex modifies the impact of the genetic variations on toxicity. TOSCA trial offered a unique opportunity for performing a sex-related pharmacogenetic study in an optimal setting where, as far as possible considering the TOSCA trial started in 2007, women and men were characterized and uniformly assessed for clinical/pathologic characteristics and the monitoring of toxicity. We introduced a time-to-event analysis for detecting pharmacogenetic associations with chemotherapy-induced adverse events. The time-to-event analysis may be useful to find potential clinical impact of polymorphisms, which could be lost in a common binary analysis of genotype frequencies in contingency table^27^. This type of analysis adds the dimensional time, it allows for detection of more and early toxicity events and may help to define the clinical impact of risk alleles.

In conclusion, sex in pharmacogenetic studies is crucial and can affect the genetic variations on gene regulation and as consequence responses to therapies. Considering that we are in the era of personalized medicine, sex (biological) and gender (psychosocial-cultural) cannot be ignored any longer.

## Supplementary information

Supplementary material

Ethic commitee list
