# Author Correction: Sex-Related Differences in Impact on Safety of Pharmacogenetic Profile for Colon Cancer Patients Treated with FOLFOX-4 or XELOX Adjuvant Chemotherapy

**DOI:** 10.1038/s41598-020-58486-6

**Published:** 2020-01-31

**Authors:** Annamaria Ruzzo, Francesco Graziano, Francesca Galli, Fabio Galli, Eliana Rulli, Sara Lonardi, Monica Ronzoni, Bruno Massidda, Vittorina Zagonel, Nicoletta Pella, Claudia Mucciarini, Roberto Labianca, Maria Teresa Ionta, Irene Bagaloni, Enzo Veltri, Pietro Sozzi, Sandro Barni, Vincenzo Ricci, Luisa Foltran, Mario Nicolini, Edoardo Biondi, Annalisa Bramati, Daniele Turci, Silvia Lazzarelli, Claudio Verusio, Francesca Bergamo, Alberto Sobrero, Luciano Frontini, Mauro Magnani

**Affiliations:** 1 Department of Biomolecular Sciences, Università degli Studi di Urbino “Carlo Bo”, Urbino, Italy; 2 Azienda Ospedaliera “Ospedali Riuniti Marche Nord”, Pesaro, Italy; 3 Laboratory of Methodology for Clinical research, Department of Oncology, Istituto di Ricerche Farmacologiche Mario Negri IRCCS, Milano, Italy; 4 Istituto Oncologico Veneto IOV – IRCCS, Padova, Italy; 5 Ospedale San Raffaele, Milano, Italy; 6 Azienda Ospedaliera Universitaria di Cagliari, P.O. Monserrato, Monserrato, Italy; 7 Azienda Ospedaliera S. Maria della Misericordia, Udine, Italy; 8 Ospedale “B. Ramazzini”, Carpi, Italy; 9 Ospedale Papa Giovanni XXIII, Bergamo, Italy; 10 Ospedale di Gaeta ASL Latina, Gaeta, Italy; 11 Ospedale degli Infermi di Biella, Biella, Italy; 12 Ospedale “Treviglio-Caravaggio”, Treviglio, Italy; 13 Azienda Ospedaliera Santa Maria degli Angeli, Pordenone, Italy; 14 Azienda Ospedaliera Ospedale “Cervesi”, Cattolica, Italy; 15 Ospedale “F. Renzetti”, Lanciano, Italy; 16 Azienda Ospedaliera Fatebenefratelli, Milano, Italy; 17 AUSL Ospedale di Ravenna, Ravenna, Italy; 18 Azienda Ospedaliera di Cremona, Cremona, Italy; 19 Ospedale di Saronno, Saronno, Italy; 20 Azienda Ospedaliera “Ospedale San Martino”, Genova, Italy; 21 Fondazione GISCAD, Vanzago, Italy

Correction to: *Scientific Reports* 10.1038/s41598-019-47627-1, published online 08 August 2019

The original version of this Article contained an error in Affiliation 4, which was incorrectly given as ‘IOV- IRCCS, Padova, Italy’. The correct affiliation is listed below:

Istituto Oncologico Veneto IOV – IRCCS, Padova, Italy

This error has now been corrected in the HTML and PDF versions of the Article, and in the accompanying Supplemental Material.

