# Supplementary material for: Sex-Related Differences in Impact on Safety of Pharmacogenetic Profile for Colon Cancer Patients Treated with FOLFOX-4 or XELOX Adjuvant Chemotherapy
Source: Sci Rep. 2019 Aug 8;9:11527. doi: 10.1038/s41598-019-47627-1 (PMC6687727; doi:10.1038/s41598-019-47627-1)
Supplement: Supplementary file 1 — Supplementary material [file 41598_2019_47627_MOESM1_ESM.pdf]

Sex-Related Differences in Impact On Safety Of Pharmacogenetic Profile For Colon Cancer Patients Treated With FOLFOX-4 or XELOX Adjuvant Chemotherapy

[1]*Annamaria Ruzzo*[§*], [2]*Francesco Graziano*[§], [3]*Francesca Galli,* [3]*Fabio Galli,* [3]*Eliana Rulli,* [4]*Sara Lonardi,* [5]*Monica Ronzoni,* [6]*Bruno Massidda,* [4]*Vittorina Zagonel,* [7]*Nicoletta Pella,* [8]*Claudia Mucciarini,* [9]*Roberto Labianca,* [6]*Maria Teresa Ionta,* [1]*Irene Bagaloni,* [10]*EnzoVeltri,* [11]*Pietro Sozzi,* [12]*Sandro Barni,* [5]*Vincenzo Ricci,* [13]*Luisa Foltran,* [14]*Mario Nicolini,* [15]*Edoardo Biondi,* [16]*Annalisa Bramati,* [17]*Daniele Turci,* [18]*Silvia Lazzarelli,* [19]*Claudio Verusio,* [4]*Francesca Bergamo,* [20]*Alberto Sobrero,* [21]*Luciano Frontini,* [1]*Mauro Magnani.*

[1]Department of Biomolecular Sciences, Università degli Studi di Urbino "Carlo Bo", Urbino, Italy. [2]Azienda Ospedaliera "Ospedali Riuniti Marche Nord", Pesaro, Italy. [3]Laboratory of Methodology for Clinical research, Department of Oncology, Istituto di Ricerche Farmacologiche Mario Negri IRCCS, Milano, Italy. [4]IOV- IRCCS, Padova, Italy. [5]Ospedale San Raffaele, Milano, Italy. [6]Azienda Ospedaliera Universitaria di Cagliari, P.O. Monserrato, Italy. [7]Azienda Ospedaliera S. Maria della Misericordia, Udine, Italy. [8]Ospedale "B. Ramazzini", Carpi, Italy. [9]Ospedale Papa Giovanni XXIII, Bergamo, Italy. [10]Ospedale di Gaeta ASL Latina, Italy. [11]Ospedale degli Infermi di Biella. [12]Ospedale "Treviglio-Caravaggio", Treviglio, Italy. [13]Azienda Ospedaliera Santa Maria degli Angeli, Pordenone, Italy. [14]Azienda Ospedaliera Ospedale "Cervesi", Cattolica, Italy. [15]Ospedale "F, Renzetti", Lanciano, Italy. [16]Azienda Ospedaliera Fatebenefratelli, Milano, Italy. [17]AUSL Ospedale di Ravenna, Ravenna, Italy. [18]Azienda Ospedaliera di Cremona, Cremona, Italy. [19]Ospedale di Saronno, Saronno, Italy. [20]Azienda Ospedaliera "Ospedale San Martino", Genova, Italy. [21]Fondazione GISCAD, Parabiago, Italy.

[§]A. Ruzzo and F. Graziano contributed equally to the study

*Annamaria Ruzzo, Department of Biomolecular Sciences (DiSB)
University of Urbino "Carlo Bo", Via Arco d'Augusto, 2 Italy 61032-FANO (PU)
Tel: +390722304957 *e-mail: annamaria.ruzzo@uniurb.it

Supplementary Tables and Figure

Table S1. Demographic and clinical characteristics. Comparison between the TOSCA trial and the ancillary study samples.

	Ancillary studyN=512	TOSCA trial N=3654	P-value
**Age**			0.9838
Mean (SD)	63.3 (9.5)	63.3 (9.7)	
Median (Q1 - Q3)	64.0 (57.4-70.7)	64.5 (57.3-70.6)	
Min - Max	25.1 - 82.3	21.0 - 83.8	
**Sex**			0.4534
Maschio	294 (57.4)	2034 (55.7)	
Femmina	218 (42.6)	1620 (44.3)	
Missing	0	3654	
**ECOG Performance status - n (%)**			0.1051*
0	494 (96.5)	3461 (94.8)	
1	18 (3.5)	189 (5.2)	
2	0 (0.0)	1 (0.0)	
Missing	0	3	
**Tumor site**			0.0323
Single site	489 (95.5)	3541 (97.2)	
Multiple site	23 (4.5)	101 (2.8)	
Missing	0	12	
**Single site specification - n (%)**			0.5615
Ascending colon	138 (28.2)	1106 (31.3)	
Hepatic flexure	27 (5.5)	150 (4.2)	
Trasverse colon	32 (6.5)	240 (6.8)	
Splenic flexure	24 (4.9)	134 (3.8)	
Descending colon	73 (14.9)	556 (15.7)	
Sigmoid colon	131 (26.8)	895 (25.3)	
Sigmoid-rectum colon	64 (13.1)	455 (12.9)	
Missing	23	118	
**Tumor site - n (%)**			0.3008
Right sides	198 (38.7)	1501 (41.3)	
Left sides	293 (57.2)	2024 (55.7)	
Multiple site	21 (4.1)	112 (3.1)	
Missing	0	17	

*Comparison was performend excluded patients with performance status equal to 2.

Table S2. Tumor characteristics. Comparison between the TOSCA trial and the ancillary study samples.

	Ancillary study N=512	TOSCA trial N=3654	P-value
**Histology - n (%)**			0.4270
Adenocarcinoma	441 (86.1)	3217 (88.2)	
Adenocarcinoma mucoide	65 (12.7)	398 (10.9)	
Carcinoma a cellule ad anello con castone	3 (0.6)	16 (0.4)	
Altro	1 (0.2)	7 (0.2)	
Carcinoma midollare	2 (0.4)	3 (0.1)	
Carcinoma adenosquamoso	0 (0.0)	4 (0.1)	
Carcinoma a cellule squamose	0 (0.0)	1 (0.0)	
Missing	0	8	
**Histology categorization - n (%)**			0.3636
Adenocarcinoma	441 (86.1)	3217 (88.2)	
Adenocarcinoma mucoide	65 (12.7)	398 (10.9)	
Altro	6 (1.2)	31 (0.9)	
Missing	0	8	
**T stage - n (%)**			0.4702
Tx	1 (0.2)	12 (0.3)	
T0	0 (0.0)	2 (0.1)	
T1	12 (2.3)	76 (2.1)	
T2a	15 (2.9)	169 (4.6)	
T2b	16 (3.1)	78 (2.1)	
T3	385 (75.2)	2710 (74.5)	
T4	83 (16.2)	590 (16.2)	
Missing	0	17	
**N stage - n (%)**			0.4740
Nx	0 (0.0)	11 (0.3)	
N0	185 (36.1)	1232 (34.0)	
N1	237 (46.3)	1748 (48.2)	
N2	90 (17.6)	637 (17.6)	
Missing	0	26	
**Clinical stage - n (%)**			0.4777
II	185 (36.1)	1262 (34.5)	
III	327 (63.9)	2392 (65.5)	
**Clinical stage subgrups - n (%)**			0.7865
II	185 (36.1)	1262 (34.7)	
III low risk	212 (41.4)	1558 (42.9)	
III high risk	115 (22.5)	815 (22.4)	
Missing	0	19	

	Ancillary study N=512	TOSCA trial N=3654	P-value
**Grade - n (%)**			0.3210
GX: non valutabile	4 (0.8)	19 (0.5)	
G1: ben differenziato	38 (7.5)	211 (5.9)	
G2: mediamente differenziato	307 (60.6)	2295 (63.8)	
G3: scarsamente differenziato	158 (31.2)	1072 (29.8)	
Missing	5	57	
**Chemotherapy taken during the TOSCA trial - n (%)**			0.0010
Folfox-4 (6 months)	186 (36.3)	1195 (32.7)	
Xelox (24 weeks)	71 (13.9)	686 (18.8)	
Folfox-4 (3 months)	187 (36.5)	1139 (31.2)	
Xelox (12 weeks)	68 (13.3)	634 (17.4)	

Note. In TOSCA trial, treatment with Xelox was allowed when the recruitment in this ancillary study was almost concluded. No difference was detected in treatment duration (3 or 6 months) between the TOSCA and ancillary samples (p=0.5866).

Subgroup	Adjusted OR (95%CI), p-value
**Heamatological toxicity (except anemia)**	
**Co-dominant model**	
**XPD rs1799793**	0.0120*
**Overall**	
GA vs GG	1.20 (0.77-1.89), 0.4172
AA vs GG	1.31 (0.69-2.49), 0.4086
**Men**	
GA vs GG	2.30 (1.20-4.41), 0.0118
AA vs GG	1.39 (0.50-3.81), 0.5275
**Women**	
GA vs GG	0.59 (0.30-1.17), 0.1321
AA vs GG	1.18 (0.47-2.95), 0.7192
**Dominant model**	
**MTHFR rs1801133**	0.0254*
Overall, CT+TT vs CC	0.96 (0.62-1.49), 0.8551
Men, CT+TT vs CC	0.61 (0.33-1.12), 0.1133
Women, CT+TT vs CC	1.71 (0.87-3.35), 0.1188
**XPD rs1799793**	0.0185*
Overall, GA+AA vs GG	1.23 (0.81-1.87), 0.3386
Men, GA+AA vs GG	2.09 (1.12-3.89), 0.0210
Women, GA+AA vs GG	0.72 (0.38-1.35), 0.3048
**Gastrointestinal toxicity**	
**Dominant model**	
**XPD rs13181**	0.0467*
Overall, TG+GG vs TT	0.85 (0.47-1.53), 0.5915
Men, TG+GG vs TT	1.65 (0.65-4.16), 0.2925
Women, TG+GG vs TT	0.48 (0.21-1.08), 0.0759
**Neurotoxicity**	
**Co-dominant model**	
**ERCC1 rs11615**	0.0518*
**Overall**	
TC vs TT	1.57 (0.94-2.62), 0.0855
CC vs TT	1.65 (0.86-3.18), 0.1342
**Men**	
TC vs TT	1.79 (0.89-3.58), 0.1004
CC vs TT	1.01 (0.41-2.48), 0.9818
**Women**	
TC vs TT	1.35 (0.62-2.94), 0.4436
CC vs TT	3.75 (1.34-10.5), 0.0119
**Recessive model**	
**ERCC1 rs11615**	0.0174*
Overall, CC vs TC+TT	1.30 (0.72-2.34), 0.3828
Men, CC vs TC+TT	0.74 (0.33-1.67), 0.4705
Women, CC vs TC+TT	3.17 (1.26-8.00), 0.0146

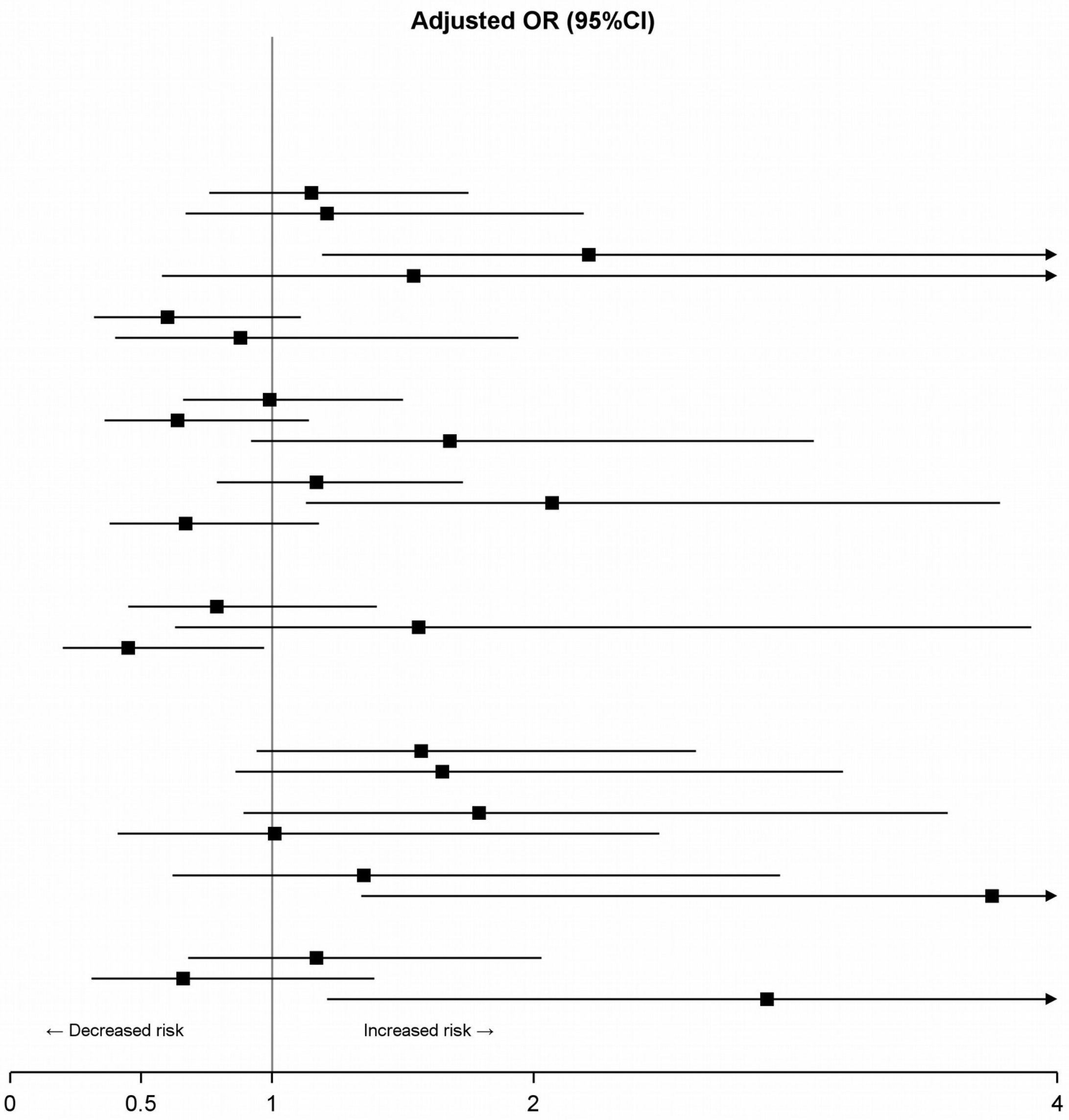

* P-value of interaction

Figure S1 – Logistic regression for time to haematological toxicity (TTH), time to gastrointestinal toxitiy (TTG) and time to neurotoxicity (TTN)
