# Supplementary material for: Sex-Related Differences in Impact on Safety of Pharmacogenetic Profile for Colon Cancer Patients Treated with FOLFOX-4 or XELOX Adjuvant Chemotherapy
Source: Sci Rep. 2019 Aug 8;9:11527. doi: 10.1038/s41598-019-47627-1 (PMC6687727; doi:10.1038/s41598-019-47627-1)
Supplement: Supplementary file 2 — Ethic commitee list [file 41598_2019_47627_MOESM2_ESM.pdf]

Sex-Related Differences in Impact On Safety Of Pharmacogenetic Profile For Colon Cancer Patients Treated With FOLFOX-4 or XELOX Adjuvant Chemotherapy

[1]Annamaria Ruzzo[§], [2]Francesco Graziano[§], [3]Francesca Galli, [3]Fabio Galli, [3]Eliana Rulli, [4]Sara Lonardi, [5]Monica Ronzoni, [6]Bruno Massidda, [4]Vittorina Zagonel, [7]Nicoletta Pella, [8]Claudia Mucciarini, [9]Roberto Labianca, [6]Maria Teresa Ionta, [1]Irene Bagaloni, [10]EnzoVeltri, [11]Pietro Sozzi, [12]Sandro Barni, [5]Vincenzo Ricci, [13]Luisa Foltran, [14]Mario Nicolini, [15]Edoardo Biondi, [16]Annalisa Bramati, [17]Daniele Turci, [18]Silvia Lazzarelli, [19]Claudio Verusio, [4]Francesca Bergamo, [20]Alberto Sobrero, [21]Luciano Frontini, [1]Mauro Magnani.*

[1]Department of Biomolecular Sciences, Università degli Studi di Urbino "Carlo Bo", Urbino, Italy. [2]Azienda Ospedaliera "Ospedali Riuniti Marche Nord", Pesaro, Italy. [3]Laboratory of Methodology for Clinical research, Department of Oncology, Istituto di Ricerche Farmacologiche Mario Negri IRCCS, Milano, Italy. [4]IOV- IRCCS, Padova, Italy. [5]Ospedale San Raffaele, Milano, Italy. [6]Azienda Ospedaliera Universitaria di Cagliari, P.O. Monserrato, Italy. [7]Azienda Ospedaliera S. Maria della Misericordia, Udine, Italy. [8]Ospedale "B. Ramazzini", Carpi, Italy. [9]Ospedale Papa Giovanni XXIII, Bergamo, Italy. [10]Ospedale di Gaeta ASL Latina, Italy. [11]Ospedale degli Infermi di Biella. [12]Ospedale "Treviglio-Caravaggio", Treviglio, Italy. [13]Azienda Ospedaliera Santa Maria degli Angeli, Pordenone, Italy. [14]Azienda Ospedaliera Ospedale "Cervesi", Cattolica, Italy. [15]Ospedale "F, Renzetti", Lanciano, Italy. [16]Azienda Ospedaliera Fatebenefratelli, Milano, Italy. [17]AUSL Ospedale di Ravenna, Ravenna, Italy. [18]Azienda Ospedaliera di Cremona, Cremona, Italy. [19]Ospedale di Saronno, Saronno, Italy. [20]Azienda Ospedaliera "Ospedale San Martino", Genova, Italy. [21]Fondazione GISCAD, Parabiago, Italy.

[§]A. Ruzzo and F. Graziano contributed equally to the study

*Annamaria Ruzzo, Department of Biomolecular Sciences (DiSB)
University of Urbino "Carlo Bo", Via Arco d'Augusto, 2 Italy 61032-FANO (PU)
Tel: +390722304957 *e-mail: annamaria.ruzzo@uniurb.it

Supplementary information: list of local Ethics Committee

1. A.O.U San Martino, Genova - Medicina Interna ad indirizzo Oncologico
2. A.O. Ospedale degli Infermi di Rimini, Oncologia ed Oncoematologia
3. A.O.U. San Martino, Genova - Oncologia Medica
4. Fondazione Poliambulanza, Brescia – Oncologia Medica
5. Ospedale Civile, Latisana - Oncologia Medica
6. A.O. S. Maria della Gruccia, Montevarchi - Oncologia Medica
7. A.O. della Valtellina e Valchiavenna, Sondrio - Oncologia Medica
8. Presidio Sanitario Gradenigo , Torino - Oncologia Medica
9. A.O. Umberto I, Nocera Inferiore – Oncoematologia
10. AUSL 4 Prato, Prato - Oncologia Medica
11. A.O. S.Camillo Forlanini - Roma - Oncologia Medica
12. A.O. Santa Maria Annunziata, Antella - Oncologia Medica
13. AUSL di Ravenna, Faenza - Oncologia Medica
14. Centro di Riferimento Oncologico Basilicata. Rionero in Vulture – Onco-ematologia
15. A.O. Alessandro Manzoni, Lecco - Oncologia Medica
16. Ospedale Civile, Sassari - Oncologia Medica
17. Ospedale Civile Renzetti, Lanciano - Oncologia Medica
18. Istituto Clinico Humanitas, Rozzano - Oncologia Medica ed Ematologia
19. A.O. Santa Chiara APSS, Trento - Oncologia Medica
20. A.O. Sant'Orsola-Fatebenefratelli, Brescia – Oncologia Medica
21. A.O. G. Rummo, Benevento – Oncologia
22. Policlinico Universitario di Cagliari - Oncologia Medica II

23. A.O.U. Vittorio Emanuele, Catania - Oncologia Medica
24. A.O. Carlo Poma, Mantova - Oncologia Medica
25. Ospedale Belcolle, Viterbo - Oncologia Medica
26. Ospedale Centrale, Bolzano - Oncologia Medica
27. A.O. Ospedale di Circolo di Busto Arsizio, Saronno - Oncologia Medica
28. Arcispedale Santa Maria Nuova, Reggio Emilia - Oncologia Medica
29. A.O. S.Salvatore, L'Aquila - Medicina Sperimentale
30. A.O. S. Croce e Carle, Cuneo - Oncologia Medica
31. AUSL di Ravenna, Ravenna - Oncologia Medica
32. A.O. San Paolo, Milano - Oncologia Medica
33. A.O. Morgagni -.Pierantoni, Meldola – Oncologia
34. Ospedale Versilia, Lido di Camaiore - Oncologia Medica
35. A.O. S.Carlo , Potenza - Oncologia Medica
36. A.O. San Giovanni Calibita Fatebenefratelli, Roma - Oncologia Medica
37. A.O. San Massimo, Penne – Oncologia
38. A.O. Ospedale Luigi Sacco, Milano - Oncologia Medica
39. Policlinico di Monza - Oncologia Medica
40. Policlinico Umberto I, Roma - Oncologia B
41. A.O. Cervesi, Cattolica - Oncologia Medica
42. ASL Latina, P.O. Gaeta - Oncologia Medica
43. Multimedica S. Maria, Castellanza - Oncologia Medica
44. Ospedale Civile, Lamezia Terme - Oncologia Medica
45. A.O. San Raffaele, Milano - Oncologia Medica
46. IRCCS Casa Sollievo della Sofferenza, S. Giovanni Rotondo – Oncologia
47. A.O. B.Ramazzini, Carpi - U.O. Medicina Oncologica
48. AUSL 6, P.O. di Livorno - Oncologia Medica
49. A.O.Santa Maria della Misericordia, Rovigo - Oncologia Medica
50. P.O. Terracina - Oncologia Medica
51. A.O. Careggi, Firenze - U.O. Oncologia Medica
52. A.O.U. di Parma, Parma – Oncologia Medica
53. Ospedale Civile, Asti - Oncologia Medica
54. A.O. Fatebenefratelli e Oftalmico, Milano – Oncologia
55. A.O.Cardarelli, Napoli – Oncologia
56. A.O. Umberto I, Altamura – Oncologia Medica
57. Casa di Cura IGEA, Milano - Oncologia Medica
58. Istituto Clinico S. Anna, Brescia - Oncologia Medica
59. AUSL di Ravenna, Lugo - Oncologia Medica
60. Policlinico Universitario di Cagliari - Oncologia Medica I
61. A.O. Spedali Civili di Brescia - Oncologia Medica
62. Ospedale di Manerbio, Manerbio - D.H. Oncologico
63. Istituto Oncologico G.Paolo II, Bari - Oncologia Medica e Sperimentale
64. A.O.U. Campus bio-medico, Roma - Oncologia Medica
65. A.O. della Valtellina e Valchiavenna, Sondalo - Day Hospital
66. A.O. Mellini di Chiari, Iseo - Oncologia Medica
67. A.O. Ospedale di Circolo e Fondazione Macchi - Oncologia Medica
68. A.O. San Giovanni Battista, Torino - Oncologia Medica 1
69. Fondazione San Raffaele - G. Giglio, Cefalu' - Oncologia Medica
70. Fondazione Maugeri, Pavia - Oncologia Medica I
71. A.O.Mazzoni, Ascoli Piceno - Oncologia Medica
72. A.O. Ospedale di Circolo di Melegnano, Gorgonzola - Oncologia Medica
73. Ospedale Civile A. Murri, Fermo - Oncologia Medica
74. A.O. G. da Saliceto, Piacenza - Oncologia Medica
75. A.O. S.Paolo, Savona - Oncologia Medica
76. Ospedale Civile Santo Spirito, Pescara - Oncologia Medica
77. Ospedale Civile, Legnano - Oncologia Medica
78. Fondazione Istituto Nazionale dei Tumori, Milano - Oncologia Medica
79. A.O. Valduce, Como – Oncoematologia
80. A.O. S. Camillo De Lellis, Rieti – Oncologia
81. Istituto Europeo di Oncologia, Milano - Dipartimento di Medicina
82. P.O. Bufalini, Cesena - Oncologia Medica
83. A.O. S.Giovanni Battista, Torino - Oncologia Medica 2
84. A.O. Bianchi-Melacrino-Morelli, Reggio Calabria - Oncologia Medica
85. Fondazione PTV Policlinico Tor Vergata, Roma - Oncologia Medica

86. Policlinico Umberto I - Roma - Oncologia C
87. Istituto per la Ricerca e la Cura del Cancro, Candiolo - Oncologia Medica ed Ematologia
88. Ospedale Sacro Cuore Don Calabria, Negrar - Oncologia Medica
89. AUSL 1 di Massa e Carrara, Carrara - Oncologia Medica
90. A.O. Ospedali Galliera, Genova - Oncologia Medica
91. A.O. Ospedale San Carlo Borromeo, Milano – Oncologia
92. A.O. Canosa di Puglia, Canosa – Medicina Interna e DH Oncologico
93. Policlinico Gianbattista Rossi, Verona - Oncologia Medica
94. A.O. Ospedale di Arezzo, Arezzo - Oncologia Medica
95. A.O. S.Anna, Como - Oncologia Medica
96. A.O. S. Maria della Misericordia, Udine – D.I.A.S.O
97. Multimedica IRCCSS, S.S.Giovanni - Oncologia Medica
98. A.O.Ospedale Maggiore della Carità', Novara – Oncologia
99. A.O. San Gerardo, Monza - Oncologia Medica
100. A.O. S. Salvatore, Pesaro - Divisione Oncologia
101. A.O. Sant' Andrea, Roma - Oncologia Medica
102. IDI IRCCS, Roma - Oncologia Medica IV Divisione
103. Ospedale degli Infermi, Biella - Oncologia Medica
104. P.O. S.Giovanni Bosco , Torino - Oncologia Medica
105. ASL 18 – P.O. San Lazzaro, Alba - Oncologia Medica
106. A.O. della provincia di Lodi, Casalpusterlengo - Oncologia Medica
107. A.O. Ospedali Riuniti di Bergamo – Oncologia Medica
108. A.O. Univ. Careggi, Firenze - Oncologia Medica
109. A.O. Santa Croce, Fano - Oncologia Medica
110. Istituti Ospitalieri di Cremona - Medicina e Oncologia Medica
111. A.O. E. Profili, Fabriano - U.O. Oncologia Medica
112. A.O.U. Federico II, Napoli - Dip. di Endocrinologia ed Oncologia Molecolare e Clinica
113. A.O. Ospedale Ca' Granda, Milano - Oncologia Medica
114. A.O. S.Maria degli Angeli, Pordenone - Oncologia Medica
115. A.O. Ospedale Treviglio-Caravaggio, Treviglio - Oncologia Medica
116. A.O.U. Ospedali Riuniti, Foggia - Oncologia Medica
117. A.O. A. Perrino, Brindisi - Oncologia Medica
118. AUSL 7, Siena - Oncologia Medica
119. Centro di Riferimento Oncologico, Aviano - Oncologia Medica B
120. A.O.U. Policlinico Paolo Giaccone, Palermo - Oncologia Medica
121. Istituto Nazionale per la ricerca sul cancro, Genova - Oncologia Medica A
122. A.O. Evangelico Valdese, Torino - Oncologia Medica
123. ASL 1 Umbria, Città di Castello - Oncologia Medica
124. A.O. Ospedali Riuniti Umberto I, Ancona - Divisione Clinica di Oncologia Medica
125. A.O. Univ. Policlinico Martino - Oncologia Medica
126. Fondazione G. Pascale, Napoli - Oncologia Medica A
127. Fondazione G. Pascale, Napoli - Oncologia Medica B
128. A.O. D. Cotugno, Napoli - Oncologia Medica
129. IRCCS Istituto Oncologico Veneto, Padova - Oncologia Medica 1
130. Casa di Cura La Maddalena, Palermo - Oncologia Medica
